# Comment on “Usability of the ROBOVID mobile app for health education about COVID-19”

**DOI:** 10.1590/1518-8345.7583.4380

**Published:** 2024-08-19

**Authors:** Hinpetch Daungsupawong, Viroj Wiwanitkit

**Affiliations:** 1.Private Academic Consultant, Phonhong, Lao People’s Democratic Republic.; 2.Center for Global Health Research, Saveetha Medical College, Saveetha Institute of Medical and Technical Sciences, Thandaram Kancheepuram, Tamil Nadu, India.

Dear Editor,

We would like to share ideas on the publication “Usability of the ROBOVID mobile app for health education about COVID-19”^1^. The study used a quantitative methodology to assess the usability of the ROBOVID mobile application for health education on COVID-19 among 21 individuals. The System Usability Scale was utilized to evaluate usability after the participants completed an electronic form. The ROBOVID program has good usability, as evidenced by the total average score of 87.3 on the questionnaire. It also received high marks for ease of memory, user pleasure, and ease of getting to know the system.

The limited sample size of 21 adults in this study represents a potential weakness in the technique. Results from a bigger sample size would be more reliable and broadly applicable. Furthermore, restricting the sample size to adults may limit the findings’ applicability to other age groups, including kids or senior citizens. In order to more accurately represent the population that would utilize the ROBOVID application for health education on COVID-19, future research might think about enlisting a wider range of individuals.

Among the questions raised by this study is whether users’ increased knowledge and behavior regarding COVID-19 are a result of the ROBOVID application’s good usability scores. Subsequent studies may examine how the application affects users’ comprehension of COVID-19 preventive strategies and their compliance with advised protocols. Furthermore, investigating user preferences and feedback regarding the application’s content and design may yield insightful information for later updates.

Future studies in this field may concentrate on assessing how well the ROBOVID application works over the long run to promote COVID-19-related health education and behavior changes. Studies with a longitudinal design could monitor how the program is used over time and evaluate how it affects users’ attitudes, actions, and knowledge. Additionally, looking at the possibility of including gamification components, tailored content, and interactive features in the application could improve user effectiveness and engagement with health education. In the end, the ROBOVID application’s sustained effectiveness in promoting health literacy and COVID-19 preventive measures may be attributed to ongoing evaluation and development.
