# Supplementary material for: Comment on “Usability of the ROBOVID mobile app for health education about COVID-19”
Source: Rev Lat Am Enfermagem. 2024 Aug 19;32:e4380. doi: 10.1590/1518-8345.7583.4380 (PMC11335070; doi:10.1590/1518-8345.7583.4380)
Supplement: Translation

# Translation: Comentários sobre “Usabilidade do aplicativo móvel ROBOVID para educação em saúde acerca da COVID-19”

**DOI:** 10.1590/1518-8345.7583.4381

**Authors:** Hinpetch Daungsupawong, Viroj Wiwanitkit

**Affiliations:** 1 Private Academic Consultant, Phonhong, República Democrática Popular do Laos.; 2 Center for Global Health Research, Saveetha Medical College, Saveetha Institute of Medical and Technical Sciences, Thandaram Kancheepuram, Tamil Nadu, Índia.

Prezada Editora,

Gostaríamos de compartilhar nossos pensamentos sobre o artigo “Usabilidade do aplicativo móvel ROBOVID para educação em saúde sobre COVID-19”^1^. O estudo utilizou metodologia quantitativa para avaliar a usabilidade do aplicativo móvel ROBOVID para educação em saúde sobre a COVID-19 entre 21 indivíduos. A *System Usability Scale* foi utilizada para avaliar a usabilidade após o preenchimento de um formulário eletrônico pelos participantes. O programa ROBOVID apresenta boa usabilidade, conforme evidenciado pela pontuação média total de 87,3 obtida no questionário. Ele também recebeu notas altas pela facilidade de memorização, satisfação do usuário e facilidade de conhecimento do sistema.

O tamanho limitado da amostra de 21 adultos utilizada neste estudo representa uma fraqueza potencial da metodologia. Os resultados obtidos em uma amostra maior seriam mais confiáveis e amplamente aplicáveis. Além disso, restringir a amostra a adultos pode limitar a aplicabilidade dos resultados a outras faixas etárias, incluindo crianças ou idosos. A fim de representar com mais precisão a população que utilizaria a aplicação ROBOVID para educação em saúde sobre a COVID-19, estudos futuros poderiam recrutar uma gama mais ampla de participantes.

Uma das questões levantadas por este estudo é se o aumento do conhecimento e a mudança do comportamento dos utilizadores em relação à COVID-19 resultam das boas pontuações de usabilidade do aplicativo ROBOVID. Estudos subsequentes poderão examinar como o aplicativo afeta a compreensão dos usuários acerca das estratégias preventivas da COVID-19 e sua adesão aos protocolos recomendados. Além disso, investigar as preferências do usuário e o feedback em relação ao conteúdo e ao design do aplicativo pode produzir informações úteis para subsidiar atualizações posteriores.

Estudos futuros nesta área podem concentrar-se na avaliação do funcionamento do aplicativo móvel ROBOVID a longo prazo para promover educação em saúde e mudanças de comportamento relacionadas à COVID-19. Estudos com desenho longitudinal poderiam monitorar como o programa é utilizado ao longo do tempo e avaliar como ele afeta as atitudes, ações e conhecimentos dos usuários. Além disso, considerar a possibilidade de incluir componentes de gamificação, conteúdo personalizado e recursos interativos no aplicativo poderia melhorar a eficácia do usuário e seu envolvimento com a educação em saúde. Em última análise, a eficácia sustentada do aplicativo móvel ROBOVID na promoção da literacia em saúde e das medidas preventivas da COVID-19 pode ser atribuída à sua avaliação e seu desenvolvimento contínuos.

# Translation: Comentarios en “Usabilidad de la aplicación móvil ROBOVID para educación en salud acerca de la COVID-19”

**DOI:** 10.1590/1518-8345.7583.4379

**Authors:** Hinpetch Daungsupawong, Viroj Wiwanitkit

**Affiliations:** 1 Private Academic Consultant, Phonhong, República Democrática Popular Lao.; 2 Center for Global Health Research, Saveetha Medical College, Saveetha Institute of Medical and Technical Sciences, Thandaram Kancheepuram, Tamil Nadu, India.

Estimada Editora,

Nos gustaría compartir nuestras ideas sobre la publicación “Usabilidad de la aplicación móvil ROBOVID para educación en salud acerca de la COVID-19”^1^. El estudio utilizó metodología cuantitativa para evaluar la usabilidad de la aplicación móvil ROBOVID para educación en salud sobre COVID-19 entre 21 personas. La *System Usability Scale* se utilizó para evaluar la usabilidad después de que los participantes completaron un formulario electrónico. El programa ROBOVID tiene una buena usabilidad, como lo demuestra la puntuación media total de 87,3 obtenida en el cuestionario. También recibió altas calificaciones por la facilidad de memorización, la satisfacción del usuario y la facilidad de conocimiento del sistema.

El tamaño limitado de la muestra de 21 adultos en este estudio representa una debilidad potencial de la metodología. Los resultados de una muestra mayor serían más fiables y ampliamente aplicables. Además, restringir el tamaño de la muestra a adultos puede limitar la aplicabilidad de los hallazgos a otros grupos de edad, incluidos niños o personas mayores. Para representar con mayor precisión a la población que utilizaría la aplicación ROBOVID para educación en salud sobre COVID-19, las investigaciones futuras podrían reclutar a una gama más amplia de participantes.

Entre las preguntas planteadas por este estudio está si el mayor conocimiento y major comportamiento de los usuarios respecto a la COVID-19 resultan de las buenas puntuaciones de usabilidad de la aplicación ROBOVID. Estudios posteriores pueden examinar cómo la aplicación afecta la comprensión de los usuarios sobre las estrategias preventivas de COVID-19 y su adhesion a los protocolos recomendados. Además, investigar las preferencias de los usuarios y los comentarios sobre el contenido y el diseño de la aplicación puede generar información valiosa para actualizaciones posteriores.

Los estudios futuros en este campo pueden concentrarse en evaluar qué tan bien funciona la aplicación ROBOVID a largo plazo para promover la educación en salud y los cambios de comportamiento relacionados con la COVID-19. Estudios con diseño longitudinal podrían monitorear cómo se utiliza el programa a lo largo del tiempo y evaluar cómo afecta las actitudes, acciones y conocimientos de los usuarios. Además, considerar la posibilidad de incluir componentes de gamificación, contenido personalizado y funciones interactivas en la aplicación podría mejorar la eficacia del usuario y su compromiso con la educación en salud. Al final, la eficacia sostenida de la aplicación ROBOVID a la hora de promover la alfabetización en salud y las medidas preventivas de la COVID-19 puede atribuirse a su evaluación y desarrollo continuos.
